# Word Error Analysis in Aphasia: Introducing the Greek Aphasia Error Corpus (GRAEC)

**DOI:** 10.3389/fpsyg.2020.01577

**Published:** 2020-08-04

**Authors:** Dimitrios Kasselimis, Maria Varkanitsa, Georgia Angelopoulou, Ioannis Evdokimidis, Dionysis Goutsos, Constantin Potagas

**Affiliations:** ^1^Neuropsychology and Language Disorders Unit, 1st Department of Neurology, Eginition Hospital, National and Kapodistrian University of Athens, Athens, Greece; ^2^Sargent College of Health and Rehabilitation Sciences, Boston University, Boston, MA, United States; ^3^Department of Linguistics, National and Kapodistrian University of Athens, Athens, Greece

**Keywords:** Greek, corpora, aphasia, errors, discourse, narration

## Introduction

Since the pioneering work of Paul Broca and Carl Wernicke, it has become clear that the interaction of aphasia research and theoretical linguistics can be beneficial for both disciplines: (1) in order to understand the nature of aphasia as a language disorder, it is crucial to understand the nature of language; its internal rules and principles, (2) linguistic analysis of aphasic speech can also provide some evidence on the relation between brain and language, (3) neurolinguistic data can be used to distinguish between competing linguistic theories, and (4) linguistic analysis of aphasic speech often leads to the design of linguistic-specific treatment programs for aphasia (for more details, see Avrutin, 2001).

One of the most exciting recent developments in linguistics has been the widespread use of electronic corpora, both as a methodology and a theoretical viewpoint on language (see e.g., McEnery and Hardie, 2012, for an overview). In parallel, in aphasia research, large-scale data collection and group studies allow generalizations about the population from which the participants have been drawn, leading to useful findings (see Grodzinsky et al., 1999) that can complement single case studies, which allow for a detailed description of aphasic speech patterns and inferences about the language system in non-brain damaged individuals (see amongst others Badecker and Caramazza, 1985; Caramazza, 1986; Caramazza and Badecker, 1991). However, recruiting patients with aphasia on a large scale is difficult. Even when permission for collecting and using data by patients with aphasia has been obtained, considerable resources are required to move patients through the steps of consenting, screening and testing. A solution to this problem could be data sharing, as is increasingly realized in recent bibliography, which has evidenced a surge in corpora of language datasets from speakers with various disorders, including aphasia, in several languages such as Dutch (Westerhout and Monachesi, 2007), Cantonese (Kong and Law, 2019), Russian (Khudyakova et al., 2016), Croatian (Kuvač Kraljević et al., 2017), and, of course, English (Mirman et al., 2010; Williams et al., 2010; MacWhinney et al., 2011; Laures-Gore et al., 2016). Despite such attempts of developing corpora widely available to researchers, the need for additional open data banks from different languages still remains. For instance, for Greek a recent study has presented a detailed methodology for the transcription and annotation of aphasic speech samples (Varlokosta et al., 2016); although the authors describe an elaborate pipeline, no data has been available yet.

Apart from the importance of data sharing discussed above, there is a methodological issue related to aphasic discourse analysis that is worth mentioning, namely, the method of eliciting a speech sample, which will be then used to evaluate a patient's linguistic competence on the basis of several indices, such as type and frequency of errors, semantic content, speech rate, mean length of utterance, etc. Given the large number of genres used in studies assessing aphasic narration ability (for an overview, see Müller et al., 2008), one must acknowledge the possible effects of the chosen elicitation task on the qualitative and quantitative characteristics of speech output (Armstrong, 2000), and, subsequently, the importance of evaluating verbal production across such genres (Armstrong et al., 2011).

Moreover, there has been a well-established tradition of comparing data from speakers with aphasia with general corpus data, used as controls for a variety of purposes (e.g., Schwartz et al., 1994; Gahl, 2002; Fraser et al., 2015). As reference corpora become widely available for many languages, including Greek (Goutsos, 2010), there is an increasing need for developing resources with specialized data from speakers with disorders.

To that end, we have developed the Greek Aphasia Error Corpus (GREAC), which is a large, searchable, web-based corpus of patients' performance on two different elicitation tasks, i.e., picture description and free narration, also including background language testing, and clinical/demographic information. The corpus is available at http://aphasia.phil.uoa.gr/, while a pilot sample of the data has been included in AphasiaBank (http://talkbank.org/AphasiaBank/).

## Compiling the GREAC

To our knowledge, this is the first publicly available corpus with data from Greek patients with aphasia. We present the first data from 50 right-handed monolingual Greek patients, with left stroke-induced aphasia, assessed at the Neuropsychology and Language Disorders Unit of the 1st Neurology Department of the National and Kapodistrian University of Athens, at Eginition Hospital. The participants (16 women) were 30–86 years old, with 4–20 years of formal schooling.

Background language testing included the Boston Diagnostic Aphasia Examination–Short Form (BDAE-SF) adapted for Greek (Goodglass and Kaplan, 1983; Tsapkini et al., 2009), and the Boston Naming Test (Kaplan et al., 1983), standardized in Greek (Simos et al., 2011), CT and/or MRI scans were obtained for each patient, and two independent neuroradiologists identified lesion sites, which were then coded according to previously reported methodology (Kasselimis et al., 2017). These reports are part of the publicly available database. At this point, the structural MRIs of the patients are not included in GRAEC. Demographic and speech sample information are shown in Table 1. Informed consent for participation in the study and publication of the data (ensuring anonymity) was obtained from all participants according to the Ethics Committee of Eginition Hospital. No individually identifying information—apart from time post onset, brain lesion loci, tests' performance, and basic demographic information, including sex, age, and years of formal schooling- about the patients is contained in the corpus, and individual patients are listed by random codes (see in Supplementary Tables 1, 2, for individual information regarding lesions and BDAE scores, respectively).

**Table 1:** Demographic and sound files information for the patients with aphasia.

**No**	**Code**	**Age**	**Education (years)**	**Sex**	**TPO (months)**	**Stroke story**							**Cookie theft picture**						
						**Number of words**	**Duration (s)**	**Number of errors**					**Number of words**	**Duration (s)**	**Number of errors**				
								**PH**	**MS**	**L**	**N**	**C**			**PH**	**MS**	**L**	**N**	**C**
1	A1	56	12	Male	16	87	143	4	6	0	0	12	39	102	2	0	0	1	0
2	A2	71	6	Female	16	439	204	9	8	6	0	15	311	203	11	2	2	0	22
3	A4	78	14	Female	40	218	152	2	0	0	0	2	379	484	2	0	4	0	12
4	A5	58	10	Male	1	165	218	2	4	0	0	1	127	207	4	4	12	0	0
5	A6	49	9	Male	21	113	101	4	38	0	6	0	74	140	8	16	2	0	2
6	A9	56	17	Male	3	154	268	34	6	0	6	2	60	103	8	2	6	0	2
7	A11	50	12	Male	20	68	233	12	6	2	16	0	88	197	26	0	0	4	2
8	A12	63	9	Male	1	536	238	4	6	4	0	2	304	183	0	4	0	0	6
9	A14	77	12	Male	1	NA	NA	NA	NA	NA	NA	NA	7	20	0	0	0	7	0
10	A15	71	8	Male	1	384	314	2	6	8	0	22	379	480	8	4	12	0	16
11	A19	64	6	Female	1	357	195	6	10	0	0	2	241	180	6	6	0	2	2
12	A20	74	12	Female	4	462	250	28	16	8	0	2	202	181	6	2	4	0	2
13	A26	67	10	Male	43	5	60	5	0	0	0	0	4	60	0	0	0	0	0
14	A29	63	6	Male	29	83	169	4	4	2	0	14	87	203	2	0	0	0	4
15	A32	73	12	Male	1	175	145	10	6	0	24	6	318	386	10	6	8	8	12
16	A33	34	9	Female	1	319	198	2	4	10	4	4	282	218	32	8	8	38	0
17	A35	86	12	Female	1	174	240	10	2	4	0	2	188	260	8	9	1	0	2
18	A37	58	6	Female	2	629	257	4	0	0	0	12	270	262	2	4	6	0	4
19	A38	72	12	Female	45	281	187	30	36	4	0	12	361	255	20	18	2	30	0
20	A42	55	12	Male	13	361	431	12	4	2	4	8	76	109	2	2	2	0	0
21	A43	45	14	Male	51	35	125	22	2	1	4	0	61	190	4	0	4	8	0
22	A46	50	6	Female	6	23	65	2	6	0	0	0	25	97	4	2	6	2	0
23	A51	79	6	Male	2	1040	480	40	50	16	10	22	329	190	16	28	28	2	6
24	A52	59	6	Male	10	8	80	0	0	0	0	0	24	160	0	0	0	0	6
25	A53	84	12	Male	1	509	310	18	22	8	34	12	624	429	26	22	10	61	16
26	A55	78	12	Female	3	328	200	8	22	4	4	8	152	100	2	0	6	6	8
27	A59	79	6	Female	6	102	83	12	2	0	0	6	93	80	8	2	0	10	0
28	A61	51	6	Male	9	173	117	2	0	2	0	6	61	60	2	0	0	0	2
29	A63	60	6	Female	1	163	98	16	12	0	0	2	74	70	4	6	2	0	2
30	A64	56	10	Male	13	5	60	0	0	0	0	0	3	60	0	3	0	0	0
31	A65	57	9	Male	20	390	325	4	3	0	0	6	130	70	1	2	1	0	3
32	A66	41	15	Male	1	43	110	4	2	0	10	0	16	68	0	2	0	0	4
33	A68	56	11	Male	45	10	60	0	4	8	0	5	6	60	2	4	0	0	6
34	A69	52	16	Female	2	269	182	34	6	0	4	4	85	120	22	10	0	0	2
35	A71	37	15	Male	1	3	60	2	2	0	0	2	5	60	2	2	2	0	4
36	A74	61	16	Male	4	499	320	16	4	0	0	2	150	90	8	2	4	0	2
37	A77	59	20	Male	1	54	85	0	0	2	2	4	49	130	4	0	0	2	18
38	A100	67	15	Female	4	142	175	32	2	8	4	0	NA	NA	NA	NA	NA	NA	NA
39	A103	30	17	Male	2	392	460	0	6	2	0	4	56	53	0	0	4	0	2
40	D1	51	10	Male	1	201	90	0	2	0	2	0	88	52	0	0	0	0	0
41	D4	62	9	Female	1	170	210	0	0	0	0	2	77	140	0	4	0	0	0
42	D6	39	14	Male	17	27	110	10	4	0	4	0	86	175	0	10	2	0	2
43	D10	54	12	Male	19	288	645	2	18	10	0	2	NA	NA	NA	NA	NA	NA	NA
44	D11	60	13	Male	4	154	190	20	14	2	4	2	107	150	14	2	0	0	0
45	D21	55	4	Male	33	44	80	0	0	0	0	2	38	105	0	0	0	0	2
46	D23	61	8	Male	2	290	132	10	10	0	6	0	154	110	4	2	2	0	2
47	D24	72	12	Male	1	10	60	0	0	0	0	4	5	60	0	0	0	0	2
48	D25	79	6	Male	1	41	170	6	8	0	6	2	91	135	20	13	2	14	10
49	D26	73	12	Male	1	494	325	10	20	4	2	0	166	140	2	10	0	2	2
50	D28	64	12	Male	1	9	60	0	0	0	2	0	29	60	3	4	2	6	2

*PH, phonological errors, MS, morpho-syntactic errors, L, lexical errors; N, neologisms, C, circumlocutions*.

At present, GREAC includes 17,507 words (counting only those produced by patients) with 2,397 annotated errors. GREAC is an on-going project, aiming at a corpus of approximately 50,000 words produced by 120 patients in the following 5 years. The data included in GREAC are derived from a thorough neuropsychological assessment, during which patients were first asked to talk about their illness in the form of a semi-prompted monolog (stroke story interview) and then describe the Cookie Theft picture (Picture Description task) from the BDAE-SF (Goodglass and Kaplan, 1983). All assessments were performed by a psychologist/clinical neuropsychologist in a quiet room at the Neuropsychology and Language Disorders Unit of Eginition Hospital. The examiner first initiated a short discussion with the patient, then proceeded to medical history taking, and explained in short the process of the neuropsychological assessment. During this initial interaction, the examiner made all possible efforts to establish Rapport, and make the patient feel comfortable. After that, the speech samples were obtained. First, for the stroke story, the examiner asked the patient to describe the story of their illness: “Please tell me what happened to you when you had the stroke.” Then, the patient was asked to describe the Cookie Theft picture: “Please look carefully and describe whatever you see happening in this image.” The first task was chosen in order to elicit more natural speech data, while picture elicitation was employed to ensure more controlled discourse samples, since participants have to generate a possible story from the picture without any additional requirements on memory. It must be noted that these two genres correspond to the first two of four suggested in the AphasiaBank protocol^1^. These are standard tasks, widely used in the literature (see Linnik et al., 2016 for an overview) and therefore have also been employed in GREAC in order to maximize the comparability and generalizability of findings.

Patients were given as much time as needed in both tasks with minimal prompting from the examiner when absolutely necessary. Furthermore, neurotypical adults performed the same tasks, with the only difference being that in the stroke story they were asked to narrate the stroke incident of another person (usually, a person with aphasia they accompany). We have already collected 50,000 words from 60 participants on these tasks, which at a later stage can be used as a reference corpus. GREAC will also include follow up data to allow for longitudinal studies investigating the nature of connected speech impairment in aphasia. The length of patients' connected speech samples ranges from 38 to 613 s. However, their actual speech is often less due to pausing and false starts. The Cookie Theft recordings range between 69 and 486 s.

Stroke Story and Picture Description tasks were audio-recorded. All collected material was orthographically transcribed and checked for accuracy by a second transcriber. Transcriptions included both patients and examiners' speech; however, the examiner did not interfere in patient's narration, except from the case that patients needed to be encouraged to continue their story. Standard spelling conventions were maintained to increase consistency. However, sometimes it was necessary to deviate from standard conventions, in order to transcribe as accurately as possible what was said, like in cases of unfinished words or neologisms. Fluency problems, voiced and unvoiced starters and fillers, pauses, repetitions, and other phenomena of spoken interaction such as noise from the outside, coughing etc. were carefully noted, following conventions for spoken data transcription (Georgakopoulou and Goutsos, 2004: vii; and for Greek: Georgakopoulou and Goutsos, 1999, p. 70–72). All interjections were also transcribed to give an indication of the effortful speech of patients with aphasia. Transcribed files were named by using the patient's code and the type of interaction (f for spontaneous data, p for picture description). Preliminary findings of the corpus have been previously presented at Actas del III Congreso Internacionalde Lingüística de Corpus (Goutsos et al., 2011a).

## Annotation for Speech Errors

The texts included in the Corpus are kept in two different formats, plain and annotated for speech errors. The typology of errors follows the standard distinction between phonological, morphological and lexical/semantic errors found in the literature (e.g., Saling, 2007; Schwartz and Dell, 2016, cf. Schwartz et al., 1994). Following the relevant bibliography we have restricted annotation to lemma level errors, omitting e.g., pronoun referent or coherence errors (see Marini et al., 2011; Harris Wright and Capilouto, 2012) (Syntactic and other sentence level errors are included in morphosyntactic errors in order to avoid unnecessary repetition, since morphosyntactic marking is obligatory in Greek). First, participants' responses were recorded and then transcribed by transcribers trained in transcribing aphasic speech samples. During error annotation, transcribers indicated all words, phrases or sentences that they found to differ from the target word, phrase or sentence expected based on the task at hand. A second check by a different researcher was then performed in order to ascertain whether the decision was correct, excluding for instance dialectal forms or other instances of variation (e.g., learned forms used by older speakers). All discrepancies were discussed and resolved.

Error classification followed, on the basis of phonological, morphological and syntactic properties of the Greek language. Error types, along with representative examples, are summarized in Table 2 (in several cases the distinction between two types of errors is impossible; in this case both types of error are annotated). Error frequencies for each patient are shown in Table 1. Further details of error annotation can be found in Goutsos et al. (2011a,b). A speech sample from the Cookie theft Picture description task, including annotations according to error types, is presented in Supplementary Table 3. Individual data on error subtypes in the present sample are provided in Supplementary Table 4.

**Table 2:** Error categories in the GRAEC.

**Category**	**Type**	**Example**
*Phonological errors*Errors affecting isolated phonemes or syllables	PH1: phoneme omission	“peni” instead of “pe*r*ni,” transl. “she takes”
	PH2: phoneme addition	“*a*xarti” instead of “xarti,” transl. “paper”
	PH3: phoneme substitution	“*g*romiko” instead of “vromiko,” transl. “dirty”
	PH4: syllable omission/addition/substitution	“eninda” instead of “e*ne*ninda,” transl. “ninety”
*Morpho-syntactic errors*Errors affecting grammatical morphemes	MS1: morpheme omission	“vrisi” instead of “*i* vrisi,” transl. “*the* tap”
	MS2: morpheme addition	not available in Greek for structural reasons
	MS3: general morpheme substitution	“plenete” transl. “washes herself” instead of “pleni,” transl. “washes”
	MS4: aspect substitution	“plini” (perfective aspect) instead of “pleni” (imperfective aspect), transl. “washes”
	MS5: tense substitution	“valo” (present) instead of “evala” (past), transl. “put”
	MS6: agreement substitution	“*mia* (feminine article) proi (neuter noun)” instead of “ena proi” (neuter article-noun), transl. “one morning”
	MS7: other morpho-syntactic errors	–
*Lexical errors*Substitution of a word by another pre-existing word	L1: substitution by a word that is similar in form	“plakaki,” transl. “tile,” instead of “neraki,” transl. “water”
	L2: substitution by a word that is similar in meaning	“ruxo,” transl. “cloth,” instead of “petseta,” transl. “towel”
	L3: substitution by a nonsimilar word	“numera,” transl. “numbers,” instead of “biskota,” transl. “biscuits”
*Neologisms*Errors in which more than half of the target word was incorrect, resulting in a non-existing word	N1: Neologisms that retain the structure of a Greek word and can be classified in terms of part of speech	“jerevitis” instead of “neroxitis,” transl. “sink”
	N2: Neologisms that are non-recognizable and unclassified words	“dilepona”
*Circumlocutions*	Phrases used instead of a target word	“afto pu exi to nero,” transl. “the one that has the water,” instead of “vrisi,” transl. “the tap”

## Contribution of the Corpus

The development of GREAC puts a much-needed emphasis on spontaneously produced data and the analysis of speech errors in their discourse context. Apart from the examination of speech errors, GREAC can be immensely helpful in the study of Greek aphasia in several other ways. First, information can be retrieved from the corpus on the frequency and types of phonological and lexical errors in Greek, including neologisms and other semantically related errors. Also, comparisons between GREAC and a reference corpus of Greek, such as the Corpus of Greek Texts (CGT, see Goutsos, 2010), or a similar corpus that contains patients' data from another language, such as the Cambridge Cookie-Theft Corpus (Williams et al., 2010), could result in interesting findings. A further interesting aspect of aphasic speech that could be explored using GREAC could be the use of combination of words or lexical bundles in terms of Biber et al. (1999). In GREAC the most frequent word combinations include phrases such as “I cannot/could not say/understand it,” “how to say it/what can I say,” “it must be,” “these things/this thing over here” (for further examples of errors, see Table 2). These findings are significant not only in revealing the discourse strategies followed by speakers with aphasia (e.g., avoidance, modality, periphrasis), but also for a further exploration of formulaic language in aphasia, which, as known, is processed in different ways than the rest of the vocabulary (e.g., Wray, 2002). More generally, extended data from aphasic discourse in languages like Greek are expected to contribute to the investigation of its linguistic properties in comparison with other languages; for example, the pilot version of GREAC has been compared to English and Hungarian data, suggesting that word frequency distribution is similar to non-aphasic discourse, whereas differences between languages can be related to languages' morphological properties and particular language impairments (Neophytou et al., 2017).

The detailed error annotation can also provide important evidence for the distribution of error types, especially the pervasive phonological vs. semantic distinction (Schuchard et al., 2017; McKinnon et al., 2018; Harvey et al., 2019), as well as of sub-categories of error types, that is the relative frequency of substitution, omission, addition etc. in order to test the findings of earlier linguistic studies of aphasic discourse (e.g., Blumstein, 1973; Lesser, 1995). More details can be obtained for e.g., the distribution of phonetic vs. phonemic errors (Ash et al., 2010), semantic errors vs. errors of omission (Bormann et al., 2008), the characteristics of errors of omission (vs. errors of commission, Chen et al., 2019), the target relatedness of neologistic errors (Pilkington et al., 2017) etc. Moreover, individual information on speech sample characteristics, such as total number of words and duration, could be used by researchers for participant selection according to specific exclusion criteria, or as covariates in statistical analyses. Finally, by relating data to metadata, including the level of severity of aphasia, GREAC can contribute to the development of a baseline for Greek for the automatic recognition of aphasic speech (cf. Le and Mower Prevost, 2016 for English).

Furthermore, the question of aphasia types can be studied on a much firmer basis. Different speech errors have been associated in the literature with different aphasia types (Goodglass, 1981). For example, errors in tense and agreement marking have been associated with non-fluent types of aphasia (e.g., Friedmann and Grodzinsky, 1997), whereas phonological errors and neologisms have been associated with fluent types of aphasia (e.g., Schwartz et al., 2004; Stenneken et al., 2008). However, group studies have shown that patients belonging to different diagnostic categories often made similar errors (e.g., Ardila and Rosselli, 1993). By keeping a separate file on metadata such as the demographic and clinical characteristics of patients, we would be able to link language problems with the clinical assessment of aphasic deficits. Thus, it would be possible to revisit the criteria of distinguishing between phenotypes of aphasia on the basis of findings from linguistic errors, instead of following the traditional taxonomy; in this sense, openly shared databases like GREAC could aid in the effort to cut the traditional aphasia classification cord, and move forward toward more progressive schemas (see also Schwartz, 1984; Caplan, 1993; Basso, 2000; Charidimou et al., 2014; Tremblay and Dick, 2016; Kasselimis et al., 2017). Finally, follow-up data would allow for longitudinal studies on the nature of connected speech impairment in different types of aphasia.

Two issues remain to be addressed. The first one is the justification of the existence of GREAC as a standalone database. There are several reasons that led us to the decision to create GREAC. First, the number of participants is much greater compared to the Greek sample included in the AphasiaBank for instance. Second, the addition of metadata is important; as stated above, apart from demographics, GRAEC includes individual scores on BDAE, as well as lesion information. The inclusion of such variables in statistical analyses could strengthen the findings of any aphasiological study that would utilize our database. Third, as data collection progresses, we will be able to add data from more patients, as well as data from follow-up assessments from patients already included in the corpus. Our Unit is mainly focusing on language disorders, and therefore several patients with aphasia are referred to us by other Units inside Eginition Hospital, but also by other collaborating clinics. Moreover, we regularly perform follow-up assessments for clinical and research purposes, i.e., monitor the course of aphasic deficits for individual patients or investigate the recovery pattern and possible predictors of recovery at the group level (see for example, our small scale study conducted a few years ago, which included data from the acute and the chronic phase: Chatziantoniou et al., 2015). Such follow-up data have already been collected, and will gradually be incorporated in GRAEC.

The second issue is that of sample size. There have been several databanks published in other disciplines, usually in the framework of large epidemiological studies, which include tens or even hundreds of thousands of participants. However, the GREAC is not an epidemiological databank. Its purpose is to make speech data from Greek patients with aphasia available to any researcher who wants to study aphasic errors in Greek language. To the best of our knowledge, aphasiological studies (usually in the field of psycho- or neuro-linguistics) presenting rather interesting results on Greek aphasia have samples that do not exceed the number of 20 participants (e.g., Stavrakaki and Kouvava, 2003; Koukoulioti and Stavrakaki, 2014). We argue that similar studies in the future would have much more robust and generalizable results by using a greater sample derived from GRAEC. Moreover, the fact that interested researchers would have the opportunity to select samples with specific characteristics on the basis of the metadata included in GRAEC, could lead to more focused studies. Considering how difficult patient recruiting is, let alone sampling that results in a homogenous group of participants, we believe that the present databank will aid researchers to save time and allocate their resources to aspects other than baseline testing, identifying patients suitable for their study, and speech data collecting.

To summarize, the GREAC is a unique data source for Greek that provides a rich resource for future research in many aspects of language deficits in aphasia. It allows for studying large amounts of naturally occurring data, by focusing on actual language use. The data included in GREAC come from conditions which are closer to conversation or natural discourse than experimental elicitation data, based on comprehension and production tests. Therefore, although they are not of the same ecological validity as data derived from natural verbal interaction, they can help us identify phenomena that could not have occurred if a more traditional experimental design was followed. It also allows for assessing “the relative probability of particular symptom patterns and their possible etiology” (Bates et al., 1987, p. 25) and statistically evaluating aspects of actual language usage (e.g., Wright et al., 2003). Thus, we can both generalize across patients' linguistic symptoms, by treating their discourse as a coherent whole, and study individual variation by setting it against the general pattern.

## Data Availability Statement

The datasets generated for this study are available on request to the corresponding author. GREAC is available at http://aphasia.phil.uoa.gr/.

## Ethics Statement

The studies involving human participants were reviewed and approved by Eginition Hospital Ethics Committee, National and Kapodistrian Athens, School of Medicine, Greece. The patients/participants provided their written informed consent to participate in this study.

## Author Contributions

DK contributed to the conceptualization and design of the study, performed clinical language testing, and wrote the manuscript. MV contributed to the conceptualization and design of the study, performed linguistic data processing, and wrote the manuscript. GA performed linguistic data processing, and revised the manuscript. IE contributed to the design of the study and revised the manuscript. DG conceived and designed the study, supervised linguistic data processing, and wrote the manuscript. CP contributed to the conceptualization and design of the study, recruited patients, supervised clinical language testing, and revised the manuscript. All authors contributed to the article and approved the submitted version.

## Conflict of Interest

The authors declare that the research was conducted in the absence of any commercial or financial relationships that could be construed as a potential conflict of interest.

## Footnotes

^1^Retrieved at http://aphasia.talkbank.org/protocol/
