# Supplementary material for: Word Error Analysis in Aphasia: Introducing the Greek Aphasia Error Corpus (GRAEC)
Source: Front Psychol. 2020 Aug 4;11:1577. doi: 10.3389/fpsyg.2020.01577 (PMC7417660; doi:10.3389/fpsyg.2020.01577)
Supplement: Supplementary file 1 [file Data_Sheet_1.docx]

**Supplementary Table 1**. Individual data for lesioned and intact brain areas.

|  | patient code | **IC** | **EC** | **GP** | **Pt** | **CNh** | **CNt** | **Th** | **SMA** | **PrG** | **Ins** | **IFG** | **MFG** | **IPL** | **STG** | **MTG** | **ITG** | **Lesion score** |
| --- | --- | --- | --- | --- | --- | --- | --- | --- | --- | --- | --- | --- | --- | --- | --- | --- | --- | --- |
| 1 | A1 | intact |  | intact |  | intact | intact | intact | intact | intact |  |  | intact |  |  | intact | intact | 6 |
| 2 | A2 | intact | intact | intact | intact | intact | intact | intact | intact |  |  |  | intact |  |  |  | intact | 6 |
| 3 | A4 | intact | intact | intact | intact | intact | intact | intact | intact | intact | intact |  |  | intact |  |  | intact | 4 |
| 4 | A5 |  | intact | intact |  | intact | intact | intact | intact | intact | intact | intact | intact | intact | intact | intact | intact | 2 |
| 5 | A6 | intact | intact | intact | intact | intact | intact | intact |  |  |  |  |  | intact | intact | intact | intact | 5 |
| 6 | A9 | intact | intact | intact |  | intact | intact | intact | intact |  |  |  |  | intact | lesion | intact | intact | 5 |
| 7 | A11 |  |  |  |  | intact | intact | intact | intact | intact |  |  | intact | intact |  |  | intact | 8 |
| 8 | A12 | Non-available MRI/CT | | | | | | | | | | | | | | | | |
| 9 | A14 | intact |  |  |  | intact | intact | intact | intact | intact |  |  | intact | intact | intact | intact |  | 6 |
| 10 | A15 | intact |  | intact | intact | intact | intact | intact | intact | intact |  | intact | intact |  | intact | intact | intact | 3 |
| 11 | A19 |  |  |  |  |  |  |  |  |  |  |  |  |  |  |  |  | 1 |
| 12 | A20 | intact |  | intact |  |  | intact | intact | intact | intact |  | intact | intact |  | intact | intact | intact | 5 |
| 13 | A26 | intact |  | intact | intact | intact | intact | intact | intact |  |  | intact | intact |  | lesion |  |  | 6 |
| 14 | A29 | intact | intact | intact | intact | intact | intact | intact | intact | intact |  |  | intact |  | lesion |  |  | 5 |
| 15 | A32 | Non-available MRI/CT | | | | | | | | | | | | | | | | |
| 16 | A33 | intact | intact | intact | intact | intact | intact | intact | intact |  | intact | intact | intact |  |  | intact | intact | 3 |
| 17 | A35 |  |  | intact | intact | intact | intact | intact | intact |  | intact | intact | intact | intact | intact | intact | intact | 3 |
| 18 | A37 | Non-available MRI/CT | | | | | | | | | | | | | | | | |
| 19 | A38 | intact | intact | intact |  |  |  | intact | intact |  |  |  | intact |  |  | intact | intact | 8 |
| 20 | A42 | intact |  | intact |  |  |  | intact | intact | intact |  |  |  |  |  |  |  | 10 |
| 21 | A43 |  |  |  |  |  |  | intact | intact |  |  |  |  |  |  |  |  | 13 |
| 22 | A46 | Non-available MRI/CT | | | | | | | | | | | | | | | | |
| 23 | A51 | intact | intact | intact | intact | intact | intact | intact | intact |  |  | intact | intact |  | intact | intact | intact | 3 |
| 24 | A52 | intact |  | intact | intact | intact | intact | intact | intact |  |  |  |  | intact |  |  | intact | 7 |
| 25 | A53 | intact |  | intact | intact | intact | intact | intact | intact |  |  | intact | intact | intact |  | intact | intact | 4 |
| 26 | A55 | intact | intact | intact | intact | intact | intact | intact | intact | intact | intact | intact | intact |  |  |  |  | 4 |
| 27 | A59 | Non-available MRI/CT | | | | | | | | | | | | | | | | |
| 28 | A61 |  |  |  |  | intact | intact | intact | intact | intact | intact | intact | intact |  | intact | intact | intact | 5 |
| 29 | A63 |  |  |  |  |  |  |  |  |  |  |  |  |  |  |  |  | 6 |
| 30 | A64 | Non-available MRI/CT | | | | | | | | | | | | | | | | |
| 31 | A65 | Non-available MRI/CT | | | | | | | | | | | | | | | | |
| 32 | A66 |  |  |  |  |  | intact | intact | intact |  |  |  |  |  |  | intact | intact | 11 |
| 33 | A68 | Non-available MRI/CT | | | | | | | | | | | | | | | | |
| 34 | A69 | Non-available MRI/CT | | | | | | | | | | | | | | | | |
| 35 | A71 |  |  |  |  |  |  |  |  |  |  |  |  |  |  |  |  | 9 |
| 36 | A74 |  |  |  |  |  |  |  |  |  |  |  |  |  |  |  |  | 1 |
| 37 | A77 |  |  |  |  |  |  |  |  |  |  |  |  |  |  |  |  | 9 |
| 38 | A100 | intact |  | intact | intact | intact | intact | intact | intact |  |  | intact | intact |  |  | intact | intact | 5 |
| 39 | A103 |  |  |  |  |  | intact | intact | intact |  |  | intact | intact | intact |  | intact | intact | 8 |
| 40 | D1 | intact |  | intact | intact | intact | intact | intact | intact | intact |  | intact | intact |  |  |  |  | 6 |
| 41 | D4 | intact | intact | intact | intact | intact | intact |  |  | intact |  |  |  | intact | intact | intact | intact | 5 |
| 42 | D6 | intact |  |  |  |  |  | intact |  |  |  |  | intact |  |  | intact | intact | 11 |
| 43 | D10 | intact | intact | intact | intact | intact | intact | intact | intact | intact | intact | intact | intact |  |  |  |  | 4 |
| 44 | D11 |  | intact | intact | intact | intact |  | intact | intact | intact | intact | intact | intact | intact | intact | intact | intact | 2 |
| 45 | D21 |  |  |  |  |  |  | intact | intact | intact |  |  |  |  |  |  |  | 13 |
| 46 | D23 | intact | intact | intact |  | intact | intact | intact | intact | intact | intact | intact | intact |  |  |  |  | 5 |
| 47 | D24 |  | intact | intact | intact | intact | intact | intact | intact | intact |  |  |  | intact | intact | intact | intact | 4 |
| 48 | D25 |  |  | intact | intact | intact | intact | intact | intact |  |  |  | intact |  |  |  |  | 9 |
| 49 | D26 | intact |  | intact | intact | intact | intact | intact |  |  |  |  |  |  |  | intact | intact | 8 |
| 50 | D28 |  |  |  |  |  |  | intact | intact | intact |  |  | intact | intact |  |  |  | 11 |
| IC: internal capsule; EC: external capsule; GP: globus pallidus; CNh: caudate nucleus head; CNt: caudate nucleus tail; SMA: supplementary motor area; PrG: precentral gyrus; IFG: inferior frontal gyrus; MFG: middle frontal gyrus; IPL: inferior parietal lobule; STG: superior temporal gyrus; MTG: middle temporal gyrus; ITG: inferior temporal gyrus; LS: lesion score. | | | | | | | | | | | | | | | | | | |

**Supplementary Table 2**. Boston Diagnostic Aphasia Examination (BDAE) individual scores.

|  |  |  |  |  |  |  | repetition | | |  | special categories screening | | | recognition | | |  | reading | | reading comprehension | |
| --- | --- | --- | --- | --- | --- | --- | --- | --- | --- | --- | --- | --- | --- | --- | --- | --- | --- | --- | --- | --- | --- |
|  | patient code | simple social responses | words comprehension | simple commands | complex ideational material | automatized sequences | words | sentences | sentences (number of words) | responsive naming | letters | numbers | colours | letters | numbers | numbers with dots | picture-word matching | words | sentences | paragraph | sentences |
| 1 | A1 | 7 | 16 | 10 | 6 | 4 | 4 | 1 | 9 | 10 | 4 | 4 | 4 | 4 | 2 | 2 | 4 | 15 | 2 | 1 | 4 |
| 2 | A2 | 6 | 14 | 9 | 4 | 3 | 4 | 1 | 8 | 6 | 1 | 2 | 3 | 4 | 2 | 2 | 4 | 12 | 1 | 0 | 1 |
| 3 | A4 | 7 | 13 | 8 | 5 | 4 | 5 | 2 | 10 | 9 | 4 | 4 | 4 | 4 | 2 | 2 | 4 | 15 | 5 | 2 | 3 |
| 4 | A5 | 7 | 16 | 10 | 6 | 4 | 5 | 2 | 10 | 10 | 4 | 4 | 4 | 4 | 2 | 2 | 4 | 15 | 5 | 3 | 3 |
| 5 | A6 | 7 | 14 | 9 | 2 | 4 | 4 | 1 | 7 | 7 | 4 | 4 | 4 | 4 | 2 | 2 | 3 | 15 | 4 | 2 | 1 |
| 6 | A9 | 7 | 16 | 10 | 6 | 4 | 5 | 2 | 10 | 9 | 4 | 4 | 4 | 4 | 2 | 2 | 4 | 14 | 2 | 3 | 4 |
| 7 | A11 | 5 | 14 | 8 | 4 | 1 | 3 | 0 | 7 | 4 | 1 | 0 | 1 | 3 | 2 | 2 | 3 | 6 | 0 | 1 | 2 |
| 8 | A12 | 7 | 15.5 | 10 | 3 | 4 | 5 | 2 | 10 | 10 | 4 | 4 | 4 | 4 | 2 | 2 | 4 | 15 | 5 | 3 | 4 |
| 9 | A14 | 0 | 10 | 5 | 3 | 0 | 0 | 0 | 0 | 0 | 0 | 0 | 0 | 3 | 0 | 1 | 0 | 0 | 0 | 0 | 0 |
| 10 | A15 | 7 | 15 | 10 | 6 | 4 | 5 | 2 | 10 | 10 | 4 | 4 | 4 | 4 | 2 | 2 | 4 | 15 | 5 | 3 | 4 |
| 11 | A19 | 7 | 15.5 | 10 | 5 | 4 | 5 | 2 | 10 | 9 | 4 | 4 | 4 | 4 | 2 | 2 | 4 | 15 | 3 | 3 | 4 |
| 12 | A20 | 7 | 16 | 9 | 4 | 4 | 5 | 2 | 10 | 10 | 4 | 4 | 4 | 4 | 2 | 2 | 4 | 15 | 2 | 3 | 3 |
| 13 | A26 | 7 | 14 | 10 | 3 | 3 | 1 | 0 | 1 | 4 | 0 | 2 | 0 | 4 | 2 | 2 | 3 | 15 | 0 | 3 | 2 |
| 14 | A29 | 2 | 10 | 1 | 0 | 3 | 0 | 0 | 0 | 0 | 1 | 0 | 0 | 4 | 1 | 0 | 1 | 0 | 0 | 0 | 0 |
| 15 | A32 | 3 | 10 | 6 | 2 | 2 | 1 | 0 | 0 | 6 | 3 | 3 | 1 | 4 | 0 | 2 | 2 | 12 | 0 | 2 | 3 |
| 16 | A33 | 7 | 16 | 10 | 3 | 4 | 5 | 2 | 10 | 10 | 4 | 4 | 4 | 4 | 2 | 2 | 4 | 15 | 2 | 3 | 4 |
| 17 | A35 | 7 | 14.5 | 2 | 2 | 4 | 5 | 1 | 9 | 10 | 4 | 3 | 4 | 4 | 2 | 2 | 4 | 15 | 4 | 2 | 3 |
| 18 | A37 | 7 | 12.5 | 9 | 3 | 4 | 5 | 2 | 10 | 10 | 3 | 4 | 1 | 4 | 2 | 2 | 2 | 7 | 0 | 0 | 0 |
| 19 | A38 | 5 | 16 | 8 | 4 | 4 | 4 | 0 | 7 | 6 | 4 | 4 | 4 | 4 | 2 | 2 | 4 | 15 | 1 | 1 | 3 |
| 20 | A42 | 6 | 12 | 9 | 6 | 4 | 5 | 2 | 10 | 7 | 4 | 4 | 4 | 4 | 2 | 2 | 4 | 15 | 5 | 3 | 4 |
| 21 | A43 | 2 | 14 | 9 | 2 | 1 | 3 | 0 | 0 | 6 | 0 | 0 | 2 | 4 | 2 | 2 | 2 | 3 | 0 | 3 | 3 |
| 22 | A46 | 5 | 11 | 2 | 1 | 3 | 2 | 1 | 4 | 2 | 3 | 2 | 0 | 4 | 2 | 2 | 3 | 2 | 0 | 3 | 2 |
| 23 | A51 | 3 | 10.5 | 2 | 0 | 4 | 2 | 0 | 4 | 4 | 2 | 4 | 4 | 4 | 2 | 2 | 3 | 15 | 3 | 0 | 1 |
| 24 | A52 | 3 | 13 | 10 | 6 | 2 | 2 | 0 | 4 | 4 | 2 | 3 | 2 | 4 | 2 | 2 | 3 | 0 | 0 | 0 | 0 |
| 25 | A53 | 6 | 15 | 8 | 4 | 4 | 5 | 2 | 9 | 2 | 3 | 4 | 4 | 4 | 2 | 2 | 3 | 14 | 4 | 3 | 3 |
| 26 | A55 | 7 | 14 | 9 | 5 | 4 | 5 | 2 | 10 | 2 | 4 | 4 | 4 | 4 | 2 | 2 | 4 | 7 | 2 | 1 | 4 |
| 27 | A59 | 3 | 9.5 | 2 | 0 | 0 | 0 | 0 | 0 | 0 | 0 | 1 | 0 | 0 | 0 | 0 | 0 | 0 | 0 | 0 | 0 |
| 28 | A61 | 7 | 14 | 6 | 6 | 4 | 5 | 2 | 10 | 9 | 4 | 4 | 3 | 4 | 2 | 2 | 3 | 15 | 3 | 3 | 2 |
| 29 | A63 | 7 | 16 | 10 | 6 | 4 | 5 | 2 | 10 | 8 | 4 | 4 | 4 | 4 | 2 | 2 | 4 | 15 | 2 | 2 | 2 |
| 30 | A64 | 7 | 15.5 | 8 | 5 | 4 | 5 | 2 | 10 | 10 | 4 | 4 | 4 | 4 | 2 | 2 | 4 | 15 | 5 | 3 | 4 |
| 31 | A65 | 7 | 16 | 10 | 5 | 4 | 5 | 2 | 10 | 5 | 4 | 4 | 4 | 4 | 2 | 2 | 4 | 15 | 4 | 2 | 4 |
| 32 | A66 | 2 | 6.5 | 1 | 0 | 4 | 0 | 0 | 0 | 0 | 0 | 0 | 0 | 0 | 0 | 0 | 0 | 0 | 0 | 0 | 0 |
| 33 | A68 | 0 | 13 | 1 | 3 | 4 | 3 | 0 | 0 | 1 | 0 | 0 | 0 | 4 | 2 | 1 | 2 | 3 | 0 | 0 | 2 |
| 34 | A69 | 6 | 15.5 | 10 | 5 | 4 | 5 | 2 | 10 | 10 | 4 | 4 | 4 | 4 | 2 | 2 | 3 | 11 | 2 | 3 | 4 |
| 35 | A71 | 0 | 5.5 | 4 | 1 | 0 | 0 | 0 | 0 | 0 | 0 | 0 | 0 | 2 | 0 | 0 | 0 | 0 | 0 | 0 | 0 |
| 36 | A74 | 7 | 16 | 10 | 5 | 4 | 5 | 2 | 10 | 10 | 4 | 4 | 4 | 4 | 2 | 2 | 4 | 15 | 5 | 3 | 4 |
| 37 | A77 | 4 | 12 | 4 | 1 | 4 | 5 | 0 | 2 | 4 | 0 | 3 | 3 | 4 | 2 | 2 | 3 | 3 | 0 | 0 | 2 |
| 38 | A100 | 7 | 16 | 10 | 5 | 4 | 5 | 1 | 9 | 10 | 4 | 4 | 4 | 4 | 2 | 2 | 4 | 15 | 5 | 3 | 4 |
| 39 | A103 | 7 | 16 | 10 | 5 | 4 | 5 | 2 | 10 | 10 | 4 | 4 | 4 | 4 | 2 | 2 | 4 | 15 | 5 | 3 | 4 |
| 40 | D1 | 7 | 15 | 10 | 5 | 4 | 4 | 2 | 10 | 10 | 4 | 4 | 4 | 4 | 2 | 2 | 4 | 15 | 5 | 3 | 4 |
| 41 | D4 | 7 | 15 | 10 | 6 | 4 | 5 | 2 | 10 | 10 | 4 | 4 | 4 | 4 | 2 | 2 | 4 | 15 | 4 | 2 | 4 |
| 42 | D6 | 4 | 15 | 5 | 5 | 1 | 2 | 0 | 4 | 4 | 0 | 2 | 1 | 4 | 2 | 2 | 4 | 2 | 0 | 3 | 3 |
| 43 | D10 | 7 | 14 | 9 | 4 | 4 | 5 | 2 | 10 | 9 | 4 | 4 | 4 | 4 | 2 | 2 | 3 | 12 | 3 | 3 | 4 |
| 44 | D11 | 7 | 15 | 10 | 5 | 4 | 5 | 1 | 9 | 8 | 4 | 4 | 4 | 4 | 2 | 2 | 4 | 15 | 4 | 3 | 4 |
| 45 | D21 | 3 | 11 | 1 | 1 | 2 | 4 | 0 | 4 | 2 | 0 | 3 | 0 | 4 | 2 | 2 | 2 | 0 | 0 | 0 | 0 |
| 46 | D23 | 7 | 13 | 10 | 4 | 4 | 5 | 2 | 10 | 8 | 4 | 4 | 4 | 4 | 2 | 2 | 4 | 15 | 5 | 3 | 4 |
| 47 | D24 | 2 | 15 | 9 | 6 | 1 | 1 | 1 | 9 | 1 | 4 | 2 | 1 | 4 | 2 | 2 | 3 | 3 | 0 | 2 | 1 |
| 48 | D25 | 5 | 12 | 3 | 1 | 4 | 1 | 0 | 4 | 2 | 3 | 3 | 3 | 4 | 2 | 2 | 3 | 8 | 1 | 1 | 2 |
| 49 | D26 | 7 | 15 | 9 | 6 | 4 | 5 | 2 | 10 | 10 | 4 | 4 | 4 | 4 | 2 | 2 | 4 | 15 | 4 | 3 | 4 |
| 50 | D28 | 4 | 12 | 0 | 1 | 1 | 4 | 1 | 0 | 0 | 3 | 0 | 1 | 4 | 2 | 2 | 1 | 12 | 1 | 2 | 1 |

**Supplementary Table 3**. Sample of individual data for lesion and intact areas and annotated script.

| **Patient ID: A9** | | | | | | | | | | | | | | | | | | | | | | | | | |
| --- | --- | --- | --- | --- | --- | --- | --- | --- | --- | --- | --- | --- | --- | --- | --- | --- | --- | --- | --- | --- | --- | --- | --- | --- | --- |
| **Demographics** | | | | | | | **Lesion loci** | | | | | | | | | | | | | | | | | | |
| Age | Gender | Years of formal schooling | | | TPO (days) | | **IC** | **EC** | **GP** | **Putamen** | **CNH** | | **CNT** | | **Thalamus** | **SMA** | **PrG** | **Insula** | | **IFG** | **MFG** | **IPL** | **STG** | **MTG** | **ITG** |
| 56 | Male | 17 | | | 85 | | intact | intact | intact | lesion | intact | | intact | | intact | intact | lesion | lesion | | lesion | lesion | intact | lesion | intact | intact |
| **BDAE** | | | Auditory comprehension: 32 | | | | Oral expression: 32 | | | | | | | Reading: 34 | | | | | | | | **BNT**: 39 | | | |
| **GRAEC** | | | | | | | | | | | | | | | | | | | | | | | | | |
| Stroke story | | | | | | | | | | | | Cookie theft picture | | | | | | | | | | | | | |
| Words: 154 | | | | Duration: 268 sec | | | | | | | | Words: 60 | | | | | | | Duration: 103 sec | | | | | | |
| TPO: Time Post Onset; IC: internal capsule; EC: external capsule; GP: Globus Pallidus; CNH: Head of the Caudate Nucleus; CNT: Tail of the Caudate Nucleus; SMA: Supplementary Motor Area; PrG: Precentral Gyrus; IFG: Inferior Frontal Gyrus; MFG: Middle Frontal Gyrus; IPL: Inferior Parietal Lobule; STG, MTG, ITG: Superior, Middle, and Inferior Temporal Gyrus; BDAE: Boston Diagnostic Aphasia Examination; BNT: Boston Naming Test.  Speech Annotation Sample from Cookie Theft Picture Task | | | | | | | | | | | | | | | | | | | | | | | | | |
| Patients’ utterance: | | | | | | εδώ είναι η *κυρί (**PH1)** που*πχιένει (**PH3)** τα πιάτα | | | | | | | | | | | | | | | | | | | |
| IPA transcription | | | | | | eðo ine i *ciri pu *pçeni ta pçata | | | | | | | | | | | | | | | | | | | |
| Target utterance: | | | | | | eðo ine i ciria pu pleni ta pçata | | | | | | | | | | | | | | | | | | | |
| Word-by-word English translation: | | | | | | here is the *lady who *washes the dishes | | | | | | | | | | | | | | | | | | | |

| **Supplementary Table 4.** Individual data on error subtypes | | | | | | | | | | | | | | | | | | | | | | | | | | | | | | | | | | | |
| --- | --- | --- | --- | --- | --- | --- | --- | --- | --- | --- | --- | --- | --- | --- | --- | --- | --- | --- | --- | --- | --- | --- | --- | --- | --- | --- | --- | --- | --- | --- | --- | --- | --- | --- | --- |
|  |  | **Phonemic Errors** | | | | | | | | **Morpho-syntactic errors** | | | | | | | | | | | | | | **Lexical errors** | | | | | | **Neologisms** | | | | **Circumlocutions** | |
|  |  | **Stroke Story** | | | | **Cookie Theft Picture** | | | | **Stroke Story** | | | | | | | **Cookie Theft Picture** | | | | | | | **Stroke Story** | | | **Cookie Theft Picture** | | | **Stroke Story** | | **Cookie Theft Picture** | | **Stroke Story** | **Cookie Theft Picture** |
|  | **Participant code** | **PH1** | **PH2** | **PH3** | **PH4** | **PH1** | **PH2** | **PH3** | **PH4** | **MS1** | **MS2** | **MS3** | **MS4** | **MS5** | **MS6** | **MS7** | **MS1** | **MS2** | **MS3** | **MS4** | **MS5** | **MS6** | **MS7** | **L1** | **L2** | **L3** | **L1** | **L2** | **L3** | **N1** | **N2** | **N1** | **N2** |  |  |
| 1 | A1 | 0 | 0 | 4 | 0 | 2 | 0 | 0 | 0 | 2 | 0 | 0 | 0 | 0 | 4 | 0 | 0 | 0 | 0 | 0 | 0 | 0 | 0 | 0 | 0 | 0 | 0 | 0 | 0 | 0 | 0 | 1 | 0 | 12 | 0 |
| 2 | A2 | 4 | 2 | 2 | 1 | 5 | 0 | 6 | 0 | 0 | 0 | 0 | 0 | 0 | 6 | 2 | 0 | 0 | 0 | 0 | 0 | 2 | 0 | 4 | 2 | 0 | 2 | 0 | 0 | 0 | 0 | 0 | 0 | 15 | 22 |
| 3 | A4 | 2 | 0 | 0 | 0 | 0 | 2 | 0 | 0 | 0 | 0 | 0 | 0 | 0 | 0 | 0 | 0 | 0 | 0 | 0 | 0 | 0 | 0 | 0 | 0 | 0 | 2 | 2 | 0 | 0 | 0 | 0 | 0 | 2 | 12 |
| 4 | A5 | 2 | 0 | 0 | 0 | 0 | 0 | 4 | 0 | 0 | 0 | 0 | 4 | 0 | 0 | 0 | 2 | 0 | 0 | 0 | 0 | 2 | 0 | 0 | 0 | 0 | 6 | 6 | 0 | 0 | 0 | 0 | 0 | 1 | 0 |
| 5 | A6 | 4 | 0 | 0 | 0 | 8 | 0 | 0 | 0 | 30 | 0 | 0 | 0 | 4 | 2 | 2 | 4 | 0 | 0 | 4 | 0 | 6 | 2 | 0 | 0 | 0 | 2 | 0 | 0 | 4 | 2 | 0 | 0 | 0 | 2 |
| 6 | A9 | 13 | 0 | 15 | 6 | 4 | 0 | 2 | 2 | 2 | 0 | 2 | 0 | 2 | 0 | 0 | 2 | 0 | 0 | 0 | 0 | 0 | 0 | 0 | 0 | 0 | 0 | 6 | 0 | 4 | 2 | 0 | 0 | 2 | 2 |
| 7 | A11 | 6 | 0 | 6 | 0 | 8 | 0 | 18 | 0 | 6 | 0 | 0 | 0 | 0 | 0 | 0 | 0 | 0 | 0 | 0 | 0 | 0 | 0 | 0 | 0 | 2 | 0 | 0 | 0 | 12 | 4 | 4 | 0 | 0 | 2 |
| 8 | A12 | 2 | 0 | 0 | 2 | 0 | 0 | 0 | 0 | 0 | 0 | 0 | 0 | 2 | 4 | 0 | 2 | 0 | 0 | 0 | 0 | 2 | 0 | 2 | 0 | 2 | 0 | 0 | 0 | 0 | 0 | 0 | 0 | 2 | 6 |
| 9 | A14 | N/A | | | | 0 | 0 | 0 | 0 | N/A | | | | | | | 0 | 0 | 0 | 0 | 0 | 0 | 0 | N/A | | | 0 | 0 | 0 | N/A | | 7 | 0 | N/A | 0 |
| 10 | A15 | 2 | 0 | 0 | 0 | 6 | 0 | 2 | 0 | 0 | 0 | 0 | 0 | 0 | 6 | 0 | 0 | 0 | 0 | 0 | 0 | 4 | 0 | 2 | 6 | 0 | 2 | 10 | 0 | 0 | 0 | 0 | 0 | 22 | 16 |
| 11 | A19 | 2 | 0 | 0 | 4 | 6 | 0 | 0 | 0 | 0 | 0 | 0 | 0 | 0 | 8 | 2 | 2 | 0 | 0 | 0 | 0 | 4 | 0 | 0 | 0 | 0 | 0 | 0 | 0 | 0 | 0 | 2 | 0 | 2 | 2 |
| 12 | A20 | 4 | 2 | 16 | 6 | 4 | 0 | 2 | 0 | 4 | 0 | 0 | 0 | 6 | 6 | 0 | 2 | 0 | 0 | 0 | 0 | 0 | 0 | 2 | 2 | 4 | 0 | 4 | 0 | 0 | 0 | 0 | 0 | 2 | 2 |
| 13 | A26 | 0 | 0 | 0 | 5 | 0 | 0 | 0 | 0 | 0 | 0 | 0 | 0 | 0 | 0 | 0 | 0 | 0 | 0 | 0 | 0 | 0 | 0 | 0 | 0 | 0 | 0 | 0 | 0 | 0 | 0 | 0 | 0 | 0 | 0 |
| 14 | A29 | 4 | 0 | 0 | 0 | 0 | 2 | 0 | 0 | 0 | 0 | 0 | 0 | 2 | 2 | 0 | 0 | 0 | 0 | 0 | 0 | 0 | 0 | 0 | 0 | 2 | 0 | 0 | 0 | 0 | 0 | 0 | 0 | 14 | 4 |
| 15 | A32 | 2 | 2 | 6 | 0 | 2 | 2 | 6 | 0 | 0 | 0 | 0 | 0 | 0 | 4 | 2 | 4 | 0 | 0 | 0 | 0 | 0 | 2 | 0 | 0 | 0 | 0 | 6 | 2 | 20 | 4 | 8 | 0 | 6 | 12 |
| 16 | A33 | 0 | 0 | 2 | 0 | 6 | 4 | 14 | 8 | 0 | 0 | 0 | 0 | 2 | 0 | 2 | 0 | 0 | 4 | 0 | 0 | 4 | 0 | 10 | 0 | 0 | 6 | 0 | 2 | 4 | 0 | 32 | 6 | 4 | 0 |
| 17 | A35 | 6 | 0 | 2 | 2 | 4 | 0 | 0 | 4 | 0 | 0 | 0 | 0 | 0 | 0 | 2 | 0 | 0 | 0 | 0 | 0 | 2 | 7 | 4 | 0 | 0 | 1 | 0 | 0 | 0 | 0 | 0 | 0 | 2 | 2 |
| 18 | A37 | 2 | 0 | 2 | 0 | 0 | 2 | 0 | 0 | 0 | 0 | 0 | 0 | 0 | 0 | 0 | 0 | 0 | 0 | 0 | 0 | 4 | 0 | 0 | 0 | 0 | 0 | 2 | 4 | 0 | 0 | 0 | 0 | 12 | 4 |
| 19 | A38 | 18 | 0 | 12 | 0 | 7 | 0 | 13 | 0 | 4 | 0 | 12 | 0 | 0 | 16 | 4 | 3 | 0 | 1 | 0 | 0 | 12 | 2 | 4 | 0 | 0 | 2 | 0 | 0 | 0 | 0 | 16 | 14 | 12 | 0 |
| 20 | A42 | 4 | 0 | 6 | 2 | 2 | 0 | 0 | 0 | 2 | 0 | 0 | 0 | 0 | 2 | 0 | 2 | 0 | 0 | 0 | 0 | 0 | 0 | 0 | 0 | 2 | 0 | 0 | 2 | 4 | 0 | 0 | 0 | 8 | 0 |
| 21 | A43 | 8 | 2 | 10 | 2 | 0 | 0 | 2 | 2 | 0 | 0 | 0 | 0 | 0 | 2 | 0 | 0 | 0 | 0 | 0 | 0 | 0 | 0 | 1 | 0 | 0 | 0 | 0 | 4 | 4 | 0 | 2 | 6 | 0 | 0 |
| 22 | A46 | 2 | 0 | 0 | 0 | 0 | 0 | 4 | 0 | 6 | 0 | 0 | 0 | 0 | 0 | 0 | 0 | 0 | 0 | 0 | 0 | 0 | 2 | 0 | 0 | 0 | 6 | 0 | 0 | 0 | 0 | 0 | 2 | 0 | 0 |
| 23 | A51 | 18 | 2 | 8 | 12 | 6 | 2 | 4 | 4 | 16 | 6 | 10 | 2 | 4 | 12 | 0 | 2 | 6 | 4 | 0 | 0 | 16 | 0 | 4 | 6 | 6 | 0 | 4 | 24 | 8 | 2 | 2 | 0 | 22 | 6 |
| 24 | A52 | 0 | 0 | 0 | 0 | 0 | 0 | 0 | 0 | 0 | 0 | 0 | 0 | 0 | 0 | 0 | 0 | 0 | 0 | 0 | 0 | 0 | 0 | 0 | 0 | 0 | 0 | 0 | 0 | 0 | 0 | 0 | 0 | 0 | 6 |
| 25 | A53 | 2 | 4 | 4 | 8 | 8 | 6 | 8 | 4 | 2 | 0 | 0 | 2 | 0 | 10 | 8 | 0 | 0 | 4 | 0 | 0 | 18 | 0 | 0 | 4 | 4 | 0 | 0 | 10 | 32 | 2 | 57 | 4 | 12 | 16 |
| 26 | A55 | 0 | 0 | 8 | 0 | 0 | 0 | 2 | 0 | 2 | 0 | 4 | 2 | 2 | 12 | 0 | 0 | 0 | 0 | 0 | 0 | 0 | 0 | 0 | 0 | 4 | 2 | 0 | 4 | 4 | 0 | 4 | 2 | 8 | 8 |
| 27 | A59 | 2 | 4 | 4 | 2 | 6 | 0 | 2 | 0 | 0 | 0 | 0 | 0 | 0 | 0 | 2 | 0 | 0 | 0 | 0 | 0 | 2 | 0 | 0 | 0 | 0 | 0 | 0 | 0 | 0 | 0 | 8 | 2 | 6 | 0 |
| 28 | A61 | 2 | 0 | 0 | 0 | 2 | 0 | 0 | 0 | 0 | 0 | 0 | 0 | 0 | 0 | 0 | 0 | 0 | 0 | 0 | 0 | 0 | 0 | 0 | 0 | 2 | 0 | 0 | 0 | 0 | 0 | 0 | 0 | 6 | 2 |
| 29 | A63 | 8 | 0 | 6 | 2 | 0 | 0 | 4 | 0 | 5 | 2 | 3 | 0 | 0 | 2 | 0 | 4 | 0 | 2 | 0 | 0 | 0 | 0 | 0 | 0 | 0 | 0 | 0 | 2 | 0 | 0 | 0 | 0 | 2 | 2 |
| 30 | A64 | 0 | 0 | 0 | 0 | 0 | 0 | 0 | 0 | 0 | 0 | 0 | 0 | 0 | 0 | 0 | 0 | 0 | 0 | 0 | 0 | 0 | 3 | 0 | 0 | 0 | 0 | 0 | 0 | 0 | 0 | 0 | 0 | 0 | 0 |
| 31 | A65 | 3 | 1 | 0 | 0 | 1 | 0 | 0 | 0 | 0 | 0 | 0 | 0 | 0 | 3 | 0 | 2 | 0 | 0 | 0 | 0 | 0 | 0 | 0 | 0 | 0 | 1 | 0 | 0 | 0 | 0 | 0 | 0 | 6 | 3 |
| 32 | A66 | 4 | 0 | 0 | 0 | 0 | 0 | 0 | 0 | 0 | 0 | 0 | 0 | 0 | 0 | 2 | 2 | 0 | 0 | 0 | 0 | 0 | 0 | 0 | 0 | 0 | 0 | 0 | 0 | 8 | 2 | 0 | 0 | 0 | 4 |
| 33 | A68 | 0 | 0 | 0 | 0 | 2 | 0 | 0 | 0 | 0 | 0 | 2 | 0 | 0 | 2 | 0 | 4 | 0 | 0 | 0 | 0 | 0 | 0 | 6 | 2 | 0 | 0 | 0 | 0 | 0 | 0 | 0 | 0 | 5 | 6 |
| 34 | A69 | 12 | 10 | 6 | 6 | 6 | 6 | 6 | 4 | 0 | 0 | 0 | 4 | 0 | 2 | 0 | 0 | 2 | 2 | 0 | 2 | 4 | 0 | 0 | 0 | 0 | 0 | 0 | 0 | 2 | 2 | 0 | 0 | 4 | 2 |
| 35 | A71 | 2 | 0 | 0 | 0 | 0 | 0 | 2 | 0 | 0 | 0 | 0 | 0 | 2 | 0 | 0 | 0 | 0 | 0 | 0 | 0 | 2 | 0 | 0 | 0 | 0 | 0 | 2 | 0 | 0 | 0 | 0 | 0 | 2 | 4 |
| 36 | A74 | 10 | 0 | 6 | 0 | 2 | 2 | 2 | 2 | 2 | 0 | 2 | 0 | 0 | 0 | 0 | 0 | 0 | 0 | 0 | 0 | 2 | 0 | 0 | 0 | 0 | 0 | 4 | 0 | 0 | 0 | 0 | 0 | 2 | 2 |
| 37 | A77 | 0 | 0 | 0 | 0 | 4 | 0 | 0 | 0 | 0 | 0 | 0 | 0 | 0 | 0 | 0 | 0 | 0 | 0 | 0 | 0 | 0 | 0 | 0 | 0 | 2 | 0 | 0 | 0 | 2 | 0 | 2 | 0 | 4 | 18 |
| 38 | A100 | 4 | 0 | 8 | 20 | N/A | | | | 0 | 0 | 0 | 0 | 0 | 2 | 0 | N/A | | | | | | | 4 | 0 | 4 | N/A | | | 2 | 2 | N/A | | 0 | N/A |
| 39 | A103 | 0 | 0 | 0 | 0 | 0 | 0 | 0 | 0 | 0 | 0 | 0 | 4 | 0 | 2 | 0 | 0 | 0 | 0 | 0 | 0 | 0 | 0 | 0 | 2 | 0 | 0 | 4 | 0 | 0 | 0 | 0 | 0 | 4 | 2 |
| 40 | D1 | 0 | 0 | 0 | 0 | 2 | 4 | 0 | 0 | 0 | 0 | 0 | 0 | 0 | 2 | 0 | 0 | 0 | 0 | 0 | 0 | 0 | 0 | 0 | 0 | 0 | 0 | 0 | 0 | 2 | 0 | 0 | 0 | 0 | 0 |
| 41 | D4 | 0 | 0 | 0 | 0 | 0 | 2 | 0 | 0 | 0 | 0 | 0 | 0 | 0 | 0 | 0 | 2 | 0 | 0 | 0 | 0 | 2 | 0 | 0 | 0 | 0 | 0 | 0 | 0 | 0 | 0 | 0 | 0 | 2 | 0 |
| 42 | D6 | 6 | 0 | 4 | 0 | 2 | 0 | 0 | 0 | 2 | 2 | 0 | 0 | 0 | 0 | 0 | 0 | 0 | 0 | 2 | 0 | 8 | 0 | 0 | 0 | 0 | 2 | 0 | 0 | 4 | 0 | 0 | 0 | 0 | 2 |
| 43 | D10 | 2 | 0 | 0 | 0 | N/A | | | | 2 | 0 | 0 | 2 | 2 | 8 | 4 | N/A | | | | | | | 0 | 8 | 2 | N/A | | | 0 | 0 | N/A | | 2 | N/A |
| 44 | D11 | 6 | 8 | 6 | 0 | 2 | 10 | 2 | 0 | 10 | 0 | 2 | 2 | 0 | 0 | 0 | 0 | 0 | 0 | 0 | 0 | 2 | 0 | 2 | 0 | 0 | 0 | 0 | 0 | 4 | 0 | 0 | 0 | 2 | 0 |
| 45 | D21 | 0 | 0 | 0 | 0 | 0 | 0 | 0 | 0 | 0 | 0 | 0 | 0 | 0 | 0 | 0 | 0 | 0 | 0 | 0 | 0 | 0 | 0 | 0 | 0 | 0 | 0 | 0 | 0 | 0 | 0 | 0 | 0 | 2 | 2 |
| 46 | D23 | 8 | 0 | 2 | 0 | 0 | 2 | 2 | 0 | 6 | 0 | 0 | 0 | 0 | 4 | 0 | 0 | 0 | 2 | 0 | 0 | 0 | 0 | 0 | 0 | 0 | 0 | 0 | 2 | 4 | 2 | 0 | 0 | 0 | 2 |
| 47 | D24 | 0 | 0 | 0 | 0 | 0 | 0 | 0 | 0 | 0 | 0 | 0 | 0 | 0 | 0 | 0 | 0 | 0 | 0 | 0 | 0 | 0 | 0 | 0 | 0 | 0 | 0 | 0 | 0 | 0 | 0 | 0 | 0 | 4 | 2 |
| 48 | D25 | 6 | 0 | 0 | 0 | 10 | 0 | 10 | 0 | 2 | 0 | 0 | 0 | 0 | 0 | 6 | 1 | 0 | 0 | 0 | 0 | 4 | 8 | 0 | 0 | 0 | 2 | 0 | 0 | 6 | 0 | 6 | 8 | 2 | 10 |
| 49 | D26 | 4 | 0 | 6 | 0 | 2 | 0 | 0 | 0 | 4 | 0 | 0 | 0 | 0 | 4 | 12 | 6 | 0 | 0 | 0 | 0 | 0 | 4 | 2 | 0 | 2 | 0 | 0 | 0 | 0 | 2 | 2 | 0 | 0 | 2 |
| 50 | D28 | 0 | 0 | 0 | 0 | 2 | 0 | 1 | 0 | 0 | 0 | 0 | 0 | 0 | 0 | 0 | 4 | 0 | 0 | 0 | 0 | 0 | 0 | 0 | 0 | 0 | 0 | 0 | 2 | 2 | 0 | 4 | 2 | 0 | 2 |
| PH: phonological errors, PH1: phoneme omission, PH2: phoneme addition, PH3: phoneme substitution, PH4: syllable omission/addition/substitution, MS: morpho-syntactic errors, MS1: morpheme omission, MS2: morpheme addition, MS3: general morpheme substitution, MS4: aspect substitution, MS5: tense substitution, MS6: agreement substitution, MS7: other morpho-syntactic errors, L: lexical errors; L1: substitution by a word that is similar in form, L2: substitution by a word that is similar in meaning, L3: substitution by a nonsimilar word, N: neologisms, N1: Neologisms that retain the structure of a Greek word and can be classified in terms of part of speech, N2: Neologisms that are non-recognizable and unclassified words, C: circumlocutions. | | | | | | | | | | | | | | | | | | | | | | | | | | | | | | | | | | | |
|  | | | | | | | | | | | | | | | | | | | | | | | | | | | | | | | | | | | |
